# Towards a translational health information language

**DOI:** 10.1186/1878-5085-5-S1-A51

**Published:** 2014-02-11

**Authors:** Amnon Shabo

**Affiliations:** 1 IBM Haifa Research Lab, Israel

## Scientific objectives

Recent calls in the EU and US for the creation of a “universal exchange language” for health information representation [1,2] seemed to overlook the contribution of translational medicine in general, and translational informatics in particular. This paper calls for the development of a ‘translational health information language’ (THIL), which could serve the translational information continuum. Its backbone spans from the biological research results and new types of raw data coming from sources like omics assays, sensors data and imaging techniques, and then clinical trials data and on to clinical data. Alongside this backbone of information there are also contributions of economic, social and psychological considerations that quite often prevent a new and successful intervention at the bedside from scaling out to the community and policy. The main assumption of this paper is that given the current diversity of such a continuum, it is not reasonable to expect a simplified exchange language that covers all portions of that continuum. Instead, THIL strives to mix & match existing and emerging languages through fundamental touch-points in order to enable the integration of patients’ data through a conceptual workflow of continuous data encapsulation & bubbling up loop [3]. These processes lead to gradual distillation of the raw data that makes the data usable and useful at the point of care, or for early detection, prevention and well-being.

## Technological approaches

The aforementioned ‘translational health information language’ (THIL) should draw on the following major efforts:

• On the research side, ISA [4] and Nano-publication [5] could be matched to provide expressive representation of research metadata and the resulting published knowledge;

• On the omics front, a paper recently published in Cell [6], presented the iPOP format that includes data from a variety of omics technologies that were successfully used for early detection and disease prevention;

• When raw data is integrated into clinical structures, it is important to include representational languages for the three pillars of structuring health information:

○ compositional language

○ constraining syntax

○ profiling methodology

Through profiling, it is possible to put together composed & constrained representational artifacts that can be sent across the wire for semantic interoperability purposes or for incorporation into the patient-centric electronic health record [8].

Regarding terminologies, it is beneficial to draw on results of the FP7 SemanticHealthNet [7] project that attempts to resolve the fuzzy relationship and overlaps between information models and ontologies, eventually harmonizing them into a coherent eHealth infostructure.

## Outlook and Expert recommendations

**Figure 1:**
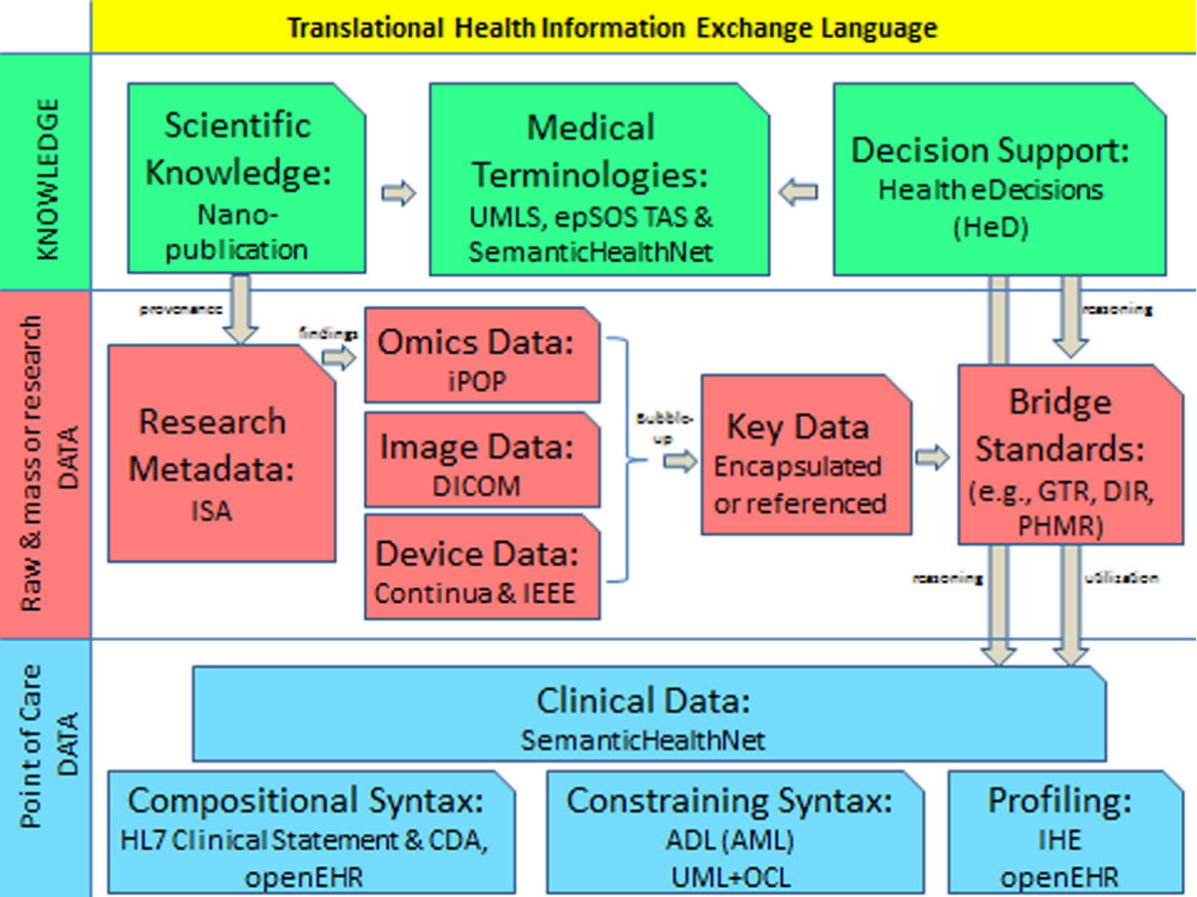
The landscape of the proposed ‘Translational Health Information Language’.

Figure 1 depicts the languages assembly proposed to be the roadmap to creating a ‘translational health information language’ (THIL). The challenges are in achieving effective touch points between the candidate schemas, for example, the Nano-publication format includes provenance as essential part of its schema, where provenance refers to a list of underpinning articles and other data sources supporting the assertion at stake. It could be much more effective if the referenced data sources are described through the ISA standard, which provides structured and unified description of the research and data used for the investigations that eventually led to the published assertion.
